# Functional Dissection of an Innate Immune Response by a Genome-Wide RNAi Screen

**DOI:** 10.1371/journal.pbio.0020203

**Published:** 2004-06-22

**Authors:** Edan Foley, Patrick H O'Farrell

**Affiliations:** **1**Department of Biochemistry and Biophysics, University of California, San FranciscoSan Francisco, CaliforniaUnited States of America

## Abstract

The innate immune system is ancient and highly conserved. It is the first line of defense and the only recognizable immune system in the vast majority of metazoans. Signaling events that convert pathogen detection into a defense response are central to innate immunity. *Drosophila* has emerged as an invaluable model organism for studying this regulation. Activation of the NF-κB family member Relish by the caspase-8 homolog Dredd is a central, but still poorly understood, signaling module in the response to gram-negative bacteria. To identify the genes contributing to this regulation, we produced double-stranded RNAs corresponding to the conserved genes in the *Drosophila* genome and used this resource in genome-wide RNA interference screens. We identified numerous inhibitors and activators of immune reporters in a cell culture model. Epistatic interactions and phenotypes defined a hierarchy of gene action and demonstrated that the conserved gene *sickie* is required for activation of Relish. We also showed that a second gene, *defense repressor 1,* encodes a product with characteristics of an inhibitor of apoptosis protein that inhibits the Dredd caspase to maintain quiescence of the signaling pathway. Molecular analysis revealed that Defense repressor 1 is upregulated by Dredd in a feedback loop. We propose that interruption of this feedback loop contributes to signal transduction.

## Abstract 2

By silencing all conserved genes in *Drosphila*, Foley and O'Farrell have identified numerous new regulators of the innate signaling response to gram-negative bacteria

## Introduction

As a typical metazoan suffers numerous microbial assaults during its lifespan, survival depends on robust defense strategies. Metazoan defenses are classified as either innate or adaptive. Adaptive immunity is characterized by elaborate genetic rearrangements and clonal selection events that produce an extraordinary diversity of antibodies and T-cell receptors that recognize invaders as nonself. While of profound importance, the adaptive responses are slow and limited to higher vertebrates. In contrast, the machinery of innate immunity is germ-line encoded and includes phylogenetically conserved signaling modules that rapidly detect and destroy invading pathogens (Medzhitov and Janeway 2000; Janeway and Medzhitov 2002). Model organisms, particularly insects, have played an important role in uncovering the wiring of innate immune pathways (Hoffmann 2003). Importantly, these organisms have provided powerful genetic approaches for identifying molecules that sense pathogens, elucidating steps that trigger innate defenses, and uncovering the weaponry used to kill or divert potential pathogens (Hoffmann et al. 1999). We have further refined the experimental approaches for rapid functional dissection of immune responses and describe new steps in an important pathway of the innate immune response.

Signaling in innate immunity consists of three steps: detection of pathogens, activation of signal transduction pathways, and mounting of appropriate defenses. The first step is triggered by the detection of pathogen-associated molecular patterns by host pattern recognition receptors (Akira et al. 2001). Typical pathogen-associated molecular patterns are β-1,3-glucan of fungi, peptidoglycan and lipopolysaccharides (LPS) of bacteria, and phosphoglycan of parasites. Signaling engages several pathways, including Toll, tumor necrosis factor, mitogen-activated protein kinase (MAPK), and Jun kinase pathways. NF-κB–type transcription factors form an important downstream nexus of the signaling pathways, and their activation promotes important defense responses. Although the defense responses are diverse and often tailored to the type of pathogen, some of the defense strategies, such as production of a panel of antimicrobial peptides, activation of phagocytic cells, and production of toxic metabolites, are evolutionarily conserved.

Interest in *Drosophila* as a model for analyzing innate immune signal transduction had a serendipitous origin. The Toll signaling pathway was discovered and characterized in *Drosophila* because of its role in specification of the embryonic dorsal ventral axis (Anderson et al. 1985). Similarities of pathway components to genes involved in mammalian immunity stimulated a hallmark study showing that the Toll pathway is a central mediator of antifungal and gram-positive bacterial defenses in *Drosophila* (Ip et al. 1993; Lemaitre et al. 1996). It is now recognized that Toll signaling is a conserved mediator of innate immune responses. A combination of classical genetics and molecular approaches has since identified numerous components of Toll signaling in *Drosophila* immunity, and it has highlighted similarities to mammals at the level of signal transduction and differences at the stage of pathogen detection (Ip et al. 1993; Rosetto et al. 1995; Nicolas et al. 1998; Drier et al. 1999; Manfruelli et al. 1999; Meng et al. 1999; Rutschmann et al. 2000a, 2002; Tauszig et al. 2000; Horng and Medzhitov 2001; Michel et al. 2001; De Gregorio et al. 2002; Ligoxygakis et al. 2002; Tauszig-Delamasure et al. 2002; Gobert et al. 2003; Weber et al. 2003).

A second pathway, the Immune deficiency (Imd) pathway, mediates responses to gram-negative bacterial infection in *Drosophila* (Lemaitre et al. 1995). Although similar to the mammalian tumor necrosis factor pathway, there are several differences between the two signaling cassettes, particularly at the level of activation. As it is presently understood, the Imd pathway is headed by an apparent pattern recognition receptor, the transmembrane peptidoglycan recognition protein LC (PGRP-LC; Choe et al. 2002; Gottar et al. 2002; Ramet et al. 2002). Although the mechanisms are largely unknown, signaling proceeds through Imd (homolog of mammalian receptor interacting protein), dTAK1 (MAP3K homolog), and a complex of Ird5/Kenny (homologous to the IKKβ/IKKγ kinase). The active IKK complex phosphorylates the p105 homolog Relish, and Dredd (caspase-8 homolog) cleaves Relish, separating an N-terminal NF-κB domain of Relish from a C-terminal ankyrin domain (Lemaitre et al. 1995; Dushay et al. 1996; Wu and Anderson 1998; Hedengren et al. 1999; Hu and Yang 2000; Leulier et al. 2000, 2002; Rutschmann et al. 2000b; Silverman et al. 2000; Stoven et al. 2000; Georgel et al. 2001; Lu et al. 2001; Vidal et al. 2001; De Gregorio et al. 2002; Gottar et al. 2002; Khush et al. 2002; Naitza et al. 2002; Silverman et al. 2003; Stoven et al. 2003; Ryu et al. 2004). The N-terminal domain enters the nucleus and promotes transcription of genes encoding proteins with defense functions such as the antimicrobial peptide Diptericin (Dipt), whose expression provides a signature for activation of the pathway.

Unlike the Toll pathway, which was thoroughly studied in its developmental capacities, analysis of the Imd pathway is relatively recent. Its more complete genetic dissection may well define another conserved and fundamental pathway of immune signaling. Of particular interest, a pivotal step in the Imd pathway—the regulation of Dredd-mediated cleavage of Relish—is not understood. To begin to address this, we developed a powerful RNA interference (RNAi)–based approach to functionally dissect the Imd pathway. In collaboration with others at the University of California, San Francisco, we produced a library of 7,216 double-stranded RNAs (dsRNAs) representing most of the phylogenetically conserved genes of *Drosophila.* We developed a cell culture assay that allowed application of this library to a high-throughput RNAi evaluation of Imd pathway activity. This screen identified numerous components of signal transduction (including negative and positive regulators of innate immune signaling), defined a hierarchy of gene action, and identified a novel gene, *sickie (sick),* required for activation of Relish. Focusing on regulation of the Dredd caspase, we identified a novel inhibitor of Dredd, Defense repressor 1 (Dnr1), which is upregulated by Dredd in a feedback loop that maintains quiescence. We propose that interruption of this feedback loop contributes to signal transduction.

## Results

### A *Drosophila* Reporter Cell Line of Imd Pathway Activity

**Figure 1:**
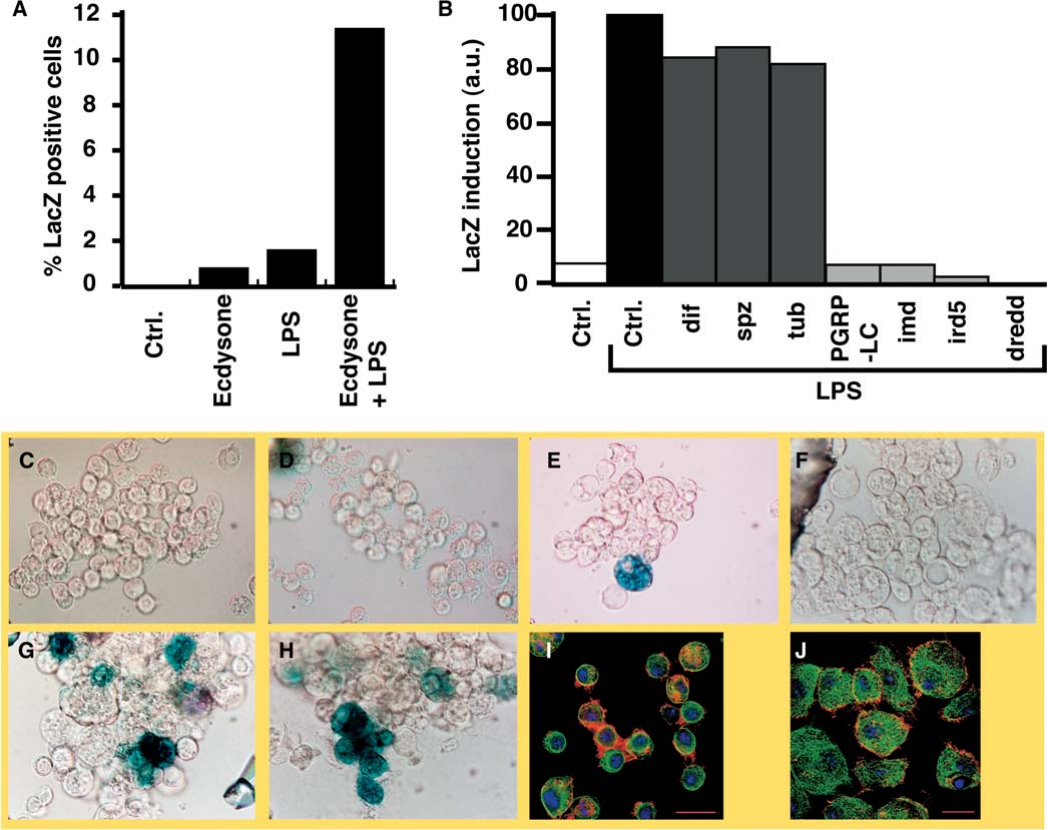
A Cell Culture Screen Identifies Novel Regulators of the Innate Immune Response (A) LPS induces an increase of about 10-fold in the number of Dipt-lacZ cells that stain positively for β-galactosidase. Ecdysone sensitizes the cells and promotes the response. (B) Dipt-lacZ induction by LPS requires known Imd signaling components, but not Tl pathway members. The fraction of β-galactosidase-positive cells was normalized to the induced control (normalized %), and influence of RNAi of Tl pathway members *(dif, spz,* and *tub)* or Imd pathway members (PGRP-LC, Imd, Ird5, and Dredd) is shown. (C–H) Activity stain (X-Gal) for β-galactosidase. (C) Untreated cells. (D) Cells treated with ecdysone alone. (E) Cells treated with ecdysone and LPS. About 10% of cells express detectable β-galactosidase. (F) RNAi against the DDRi *sick* reduces Dipt-lacZ expression in response to LPS. (G) RNAi of a representative EDRi, the Ras signaling pathway component Cnk, enhances Dipt-lacZ induction by LPS. (H) RNAi of a representative CDRi, the actin regulator SCAR induces Dipt-lacZ in the absence of LPS. (I–J) Immunofluorescence of S2 cells with actin in red, tubulin in green, and DNA in blue. Scale bars in (I) and (J) indicate 10 μm. (I) Wild-type cells have a characteristic rounded morphology. (J) RNAi against many CDRi genes disrupts morphological features of wild-type S2 cells. S2 cells are shown treated with MESR4 dsRNA. Cells are significantly larger in appearance and less round, with irregular tubulin and actin networks.

To facilitate rapid dissection of Imd pathway signaling, we established an S2 reporter cell line that expresses β-galactosidase under control of the promoter from a gene, *Dipt,* that encodes an antimicrobial peptide, Dipt-lacZ. Commercial preparations of LPS contain bacterial cell wall material capable of activating the receptor PGRP-LC and act as gratuitous inducers of antimicrobial peptide genes in *Drosophila* tissue culture cells (Samakovlis et al. 1992; Engstrom et al. 1993; Dimarcq et al. 1997). Consistent with previous studies, 20-hydroxyecdysone enhanced Dipt-lacZ induction by LPS (Figure 1A; Silverman et al. 2000, Silverman et al. 2003). Inactivation of critical Imd pathway members (PGRP-LC, Imd, Ird5, and Dredd) by RNAi virtually eliminated Dipt-lacZ induction by LPS (Figure 1B). In contrast, inactivation of the Toll pathway members Spaetzle, Tube, or Dif by RNAi had no effect on LPS-dependent induction of Dipt-lacZ. We conclude that LPS-dependent induction of Dipt-lacZ requires an intact Imd signaling pathway.

To identify additional modulators of Dipt-lacZ expression, we prepared a library of 7,216 dsRNAs representing most of the phylogenetically conserved genes of *Drosophila.* Using the Dipt-lacZ cell line, we performed a high-throughput RNAi screen for genes whose inactivation impinges on Dipt-lacZ induction. In one screen, we identified dsRNAs that altered Dipt-lacZ induction by LPS, either enhancing or suppressing activation. In a second screen performed without addition of LPS, we identified genes whose inactivation spontaneously activated the reporter. The phenotypes defined three categories of genes, which we named—based on the phenotype of their inactivation—decreased defense by RNAi (DDRi) genes, enhanced defense by RNAi (EDRi) genes, and constitutive defense by RNAi (CDRi) genes (Figure 1).

### Identification of DDRi, EDRi, and CDRi Genes

**Figure 2:**
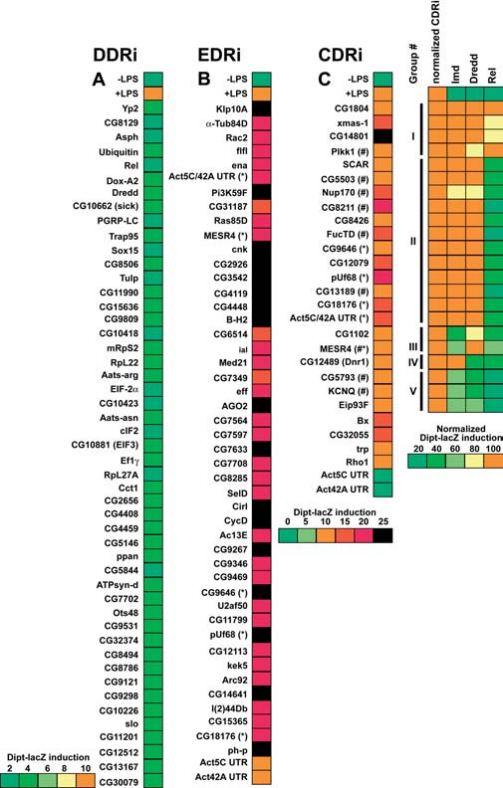
List of Modulators of the Immune Response and a False Color Display of Their Influence on Dipt-lacZ Induction The genes identified as DDRi (A), EDRi (B), and CDRi (C) are listed, and the colored bars show the influence of the corresponding dsRNA on Dipt-lacZ expression. The top two entries in (A), (B), and (C) show control cells (no dsRNA) without and with LPS, respectively. The scales for the false colors are given at the bottom left. Dipt-lacZ levels are given in terms of percent positive cells. For exact Dipt-lacZ expression values for each dsRNA refer to accompanying supplemental tables. In (B), the color scale (right) is compressed and extended compared to (A), and an asterisk indicates genes that also caused a CDRi phenotype. In (C), the pound sign indicates morphological defects and an asterisk indicates genes that also caused an EDRi phenotype, and the division of the genes into epistatic groups is shown. To the immediate left a false-color bar (coded as in [B]) indicates the effect of the dsRNAs on Dipt-lacZ expression without LPS addition. The block of colored columns shows the results of epistasis tests. Here, we set the undisturbed level of CDRi activation to 100% (as indicated in the left column in this group and the color code below), and to the right we represent reduction of this activation by prior RNAi of different Imd pathway genes. Five epistatic clusters (I–V) were identified (indicated by the lines to the left).

In an initial visual screen, dsRNAs that altered the induced or constitutive expression of β-galactosidase were selected as candidate innate immunity genes. We subjected all the initial positives to a more stringent retest where we resynthesized the candidate dsRNAs, retested these under identical conditions, and counted the number of β-galactosidase-positive cells. We defined DDRi dsRNAs as reducing the frequency of Dipt-lacZ-expressing cells to below 40% of LPS-treated controls, EDRi dsRNAs as increasing the frequency of Dipt-lacZ-expressing cells more than 2-fold, and CDRi dsRNAs as inducing Dipt-lacZ-expressing cells to a level equal to or higher than that induced by LPS. About 50% of the initial positives met these criteria, yielding 49 DDRi dsRNAs, 46 EDRi dsRNAs, and 26 CDRi dsRNAs (Figure 2A–Figure 2C; Table 1). The entire process of screening and retesting was performed without knowing the identity of the dsRNAs. Nonetheless, we successfully identified all of the known Imd pathway components in the library (PGRP-LC, Dredd, and Relish) as DDRi genes, supporting the validity of this approach for identifying genes that affect Imd pathway signaling.

**Table 1:**
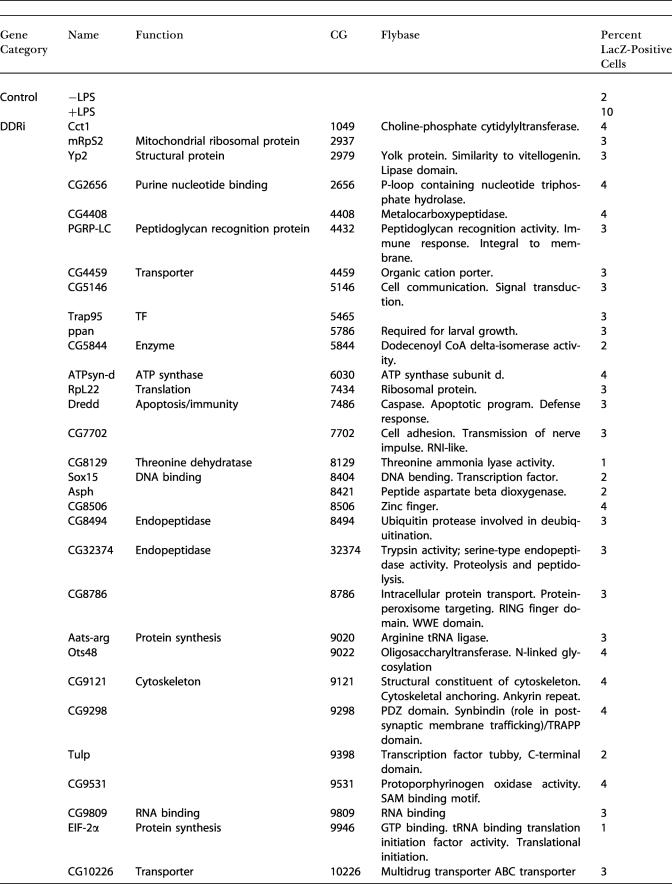
Measurement of the Percent of LacZ-Positive Cells After Treatment with dsRNA Against Individual EDRi, DDRi, and CDRi Genes

TF, transcription factor

Cell culture conditions were as described above, with the exception that CDRi genes were not treated with LPS. For each sample, 350–550 cells were counted. Controls (+ LPS and −LPS) are the average of five independent experiments. EDRi genes were defined as having greater than or equal to twice +LPS control induction levels. DDRi genes were defined as having less than or equal to 40% of +LPS control induction levels. CDRi genes were defined as having greater than or equal to four times −LPS control levels. Results were reproducible for all genes subjected to further analysis

The dsRNAs that enhance, and those that constitutively activate, the immune reporter are both expected to target inhibitors of the immune response. Nonetheless, there was only a small overlap between the EDRi genes and CDRi genes. Of the 46 confirmed EDRi dsRNAs, only five caused a CDRi phenotype, suggesting that the mechanisms that silence Imd pathway activity in the absence of infection are largely distinct from those moderating or downregulating the response to infection. We distinguish the five EDRi genes capable of constitutive activation and designate them EDRi^C^. EDRi^C^ genes are listed as both EDRi and CDRi (Figure 2B and Figure 2C, indicated with an asterisk). Approximately half of the CDRi dsRNAs also caused morphological defects (Figure 2C, indicated with a pound sign), i.e., enlarged cells with irregular cytoskeletal structures (see Figure 1J). While we do not know the basis for the altered morphology, gene expression profiling showed that LPS induces numerous cytoskeletal regulators, suggesting that cytoskeletal rearrangement is a component of the innate immune response (Boutros et al. 2002).

We also observed EDRi and CDRi phenotypes upon inactivation of Act5C and Act42A. Due to extensive sequence homology, RNAi against either actin triggers destruction of both transcripts (A. Echard, G. R. X. Hickson, E. Foley, and P. H. O'Farrell, unpublished data). Inactivation of either actin with dsRNA directed to the actin UTRs demonstrated that both actin transcripts must be inactivated for an observable EDRi or CDRi phenotype (Figure 2B and Figure 2C).

### Epistatic Evaluation of CDRi Genes

As RNAi of CDRi genes leads to ectopic Dipt-lacZ induction, we reasoned that CDRi genes are required to maintain quiescence in the absence of LPS and that induction by a CDRi dsRNA corresponds to release of inhibition of the Imd pathway. The large number of CDRi genes makes it likely that individual CDRi genes inhibit distinct steps in the Imd pathway. We sought to determine the position at which the individual CDRi genes impact the Imd pathway. In contrast to *Caenorhabditis elegans,* several genes can be inactivated by RNAi in *Drosophila* without an obvious drop in the efficiency of gene inactivation (Li et al. 2002; Schmid et al. 2002). The ability to inactivate two different gene products in sequence by RNAi provides a powerful tool to position CDRi genes relative to known Imd pathway components. In a first step, we inactivated one of three known Imd signaling components—either Imd, Dredd, or Relish. In a second step, we inactivated individual CDRi genes and monitored Dipt-lacZ induction. We reasoned that inactivation of Imd, Dredd, or Relish would not block pathway derepression by a CDRi dsRNA if the cognate CDRi impinged on the pathway at a step beyond the actions of Imd, Dredd, or Relish. Using this approach, we subdivided 20 CDRi genes into five epistatic groups (Figure 2C; Table 2). Group I contained four CDRi dsRNAs whose action was independent of Imd, Dredd, and Relish. Group II contained 12 dsRNAs whose CDRi phenotype was independent of Imd and Dredd, but depended on Relish. Group III contained two dsRNAs whose CDRi phenotype was Dredd-independent, but was reduced in the absence of Imd and Relish. Group IV contained a single dsRNA whose phenotype was independent of Imd, but dependent on Dredd and Relish. Finally, Group V contained three dsRNAs whose ability to activate the immune reporter depended on Imd, Dredd, and Relish. The epistatic relationships demonstrate that genes in Groups II–V have inputs into the known Imd pathway, while Group I might have inputs in independent pathways required for effective Dipt-lacZ expression.

**Table 2:**
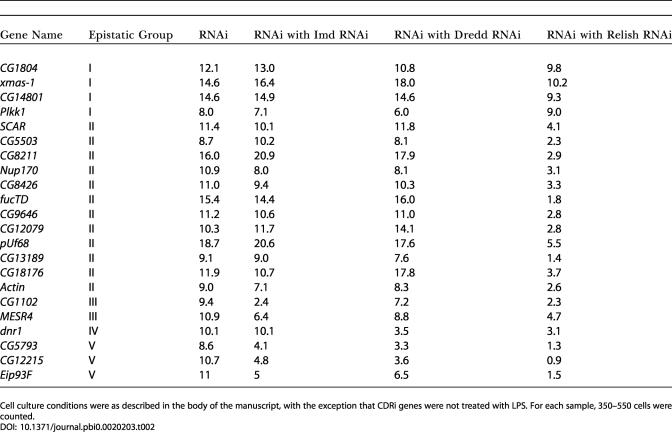
Measurement of the Percent of LacZ-Positive Cells after Treatment with dsRNA against Imd, Dredd, or Relish Followed by RNAi for the Individual CDRi

Cell culture conditions were as described in the body of the manuscript, with the exception that CDRi genes were not treated with LPS. For each sample, 350–550 cells were counted

### *Sick* Is a Conserved Gene Required for Relish Activation

**Figure 3:**
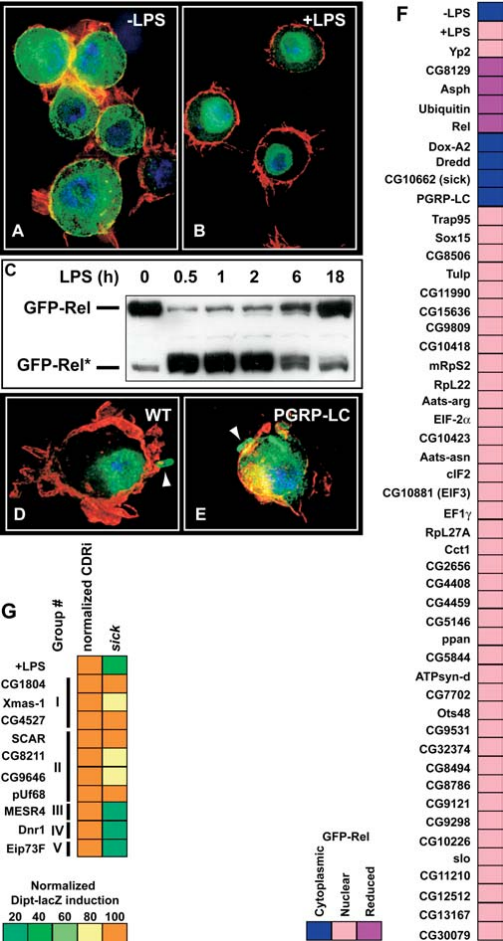
A GFP-Relish Reporter Cell Line Subdivides DDRi dsRNA into Three Categories (A–B) Immunofluorescence of GFP-Relish cells with GFP-Relish in green, DNA in blue, and actin in red. Relish is predominantly cytoplasmic in untreated control cells and rapidly translocates to the nucleus of cells incubated with LPS. (C) An anti-GFP Western blot of lysates harvested from GFP-Relish cells treated with LPS for different periods. GFP-Relish rapidly shifts from a full-length form to a shorter processed form after exposure to LPS, and full-length Relish gradually reaccumulates. (D–E) Immunohistochemistry of GFP-Relish cells incubated with GFP-expressing E. coli (arrowheads) and treated with (E) or without (D) dsRNA against PGRP-LC. Imd pathway inactivation prevents bacterial-induced Relish nuclear translocation. (F) Shows effects of treatment of GFP-Relish cells with DDRi dsRNAs for 4 d prior to LPS treatment. GFP-Relish was scored as cytoplasmic (uninduced), nuclear (induced), or reduced in amount (abnormal). (G) Shows an epistatic analysis of the DDRi, *sick.* Suppression of *sick* interferes with Dipt-lacZ induction by Group III, IV, and V CDRi dsRNAs, but not those of Groups I and II, suggesting that Sick acts downstream of Imd and Dredd, but upstream of Relish in signal transduction.

We are particularly interested in regulators contributing to activation of the Relish transcription factor by the caspase Dredd, because this is such a pivotal step in the Imd pathway and its regulation is not understood. To identify regulators that affect Relish processing, we developed an assay that more directly monitored Relish activation. We produced an S2 cell line that expresses a copper-inducible N-terminal green fluorescent protein (GFP)–tagged Relish (GFP-Relish; Figure 3). GFP-Relish is predominantly cytoplasmic in untreated cells (Figure 3A) and rapidly translocates to the nucleus upon treatment of cells with LPS or exposure to Escherichia coli (Figure 3B and Figure 3D). Western blot analysis with a monoclonal anti-GFP antibody showed that GFP-Relish is rapidly processed from a full-length form to a shorter form after exposure to LPS (Figure 3C). These findings indicate that the GFP-Relish cell line is a reliable reporter for Relish activation. Additionally, inactivation of PGRP-LC by RNAi prevented nuclear translocation of GFP-Relish in response to bacterial exposure (Figure 3E), indicating that the reporter can be used to assay function of Imd pathway genes.

We tested all DDRi dsRNAs for their effects on the response of GFP-Relish to LPS (Figure 3F). Most DDRi dsRNAs did not affect GFP-Relish levels or its LPS-stimulated nuclear concentration, suggesting that their effects on Dipt-lacZ are independent of this step of Relish activation. Four DDRi dsRNAs (Relish, ubiquitin, CG8129, and Asph) severely reduced GFP-Relish levels, indicating that these dsRNAs directly or indirectly interfered with Relish expression or stability. The ability of these dsRNAs to block Dipt-lacZ induction suggests that Dipt-lacZ induction requires substantial levels of Relish. We identified four DDRi dsRNAs that prevented LPS-stimulated nuclear translocation of GFP-Relish: PGRP-LC, Dredd, Dox-A2, and CG10662. We named CG10662 *sick*. While prolonged Dox-A2 RNAi caused cell lethality, cell viability appeared unaffected by *sick* RNAi for up to 8 d. As *sick* RNAi prevents nuclear translocation of GFP-Relish and decreases Dipt-lacZ induction after LPS treatment, we propose that the Imd pathway requires *sick* activity for Relish-dependent Dipt-lacZ induction.

Epistasis provides a second approach for positioning a DDRi gene in the hierarchy of gene action. To this end we assessed the relationship of *sick* to the five CDRi epistatic groups that we defined (above). We inactivated *sick* by RNAi and subsequently tested dsRNAs representing the five CDRi epistatic subgroupings for their ability to activate Dipt-lacZ expression in the absence of Sick (Figure 3G; Table 3). Group I and II CDRi do not require Sick, indicating that Sick acts upstream of, or in parallel to, their action, which is at the level of Relish or downstream of Relish. Induction of Dipt-lacZ by Group III and IV CDRi dsRNAs requires Sick, suggesting that Sick is required for the effective induction of Dipt-lacZ by Dredd and Imd. Combined with the observed Sick requirement for Dipt-lacZ induction and the nuclear translocation of Relish by LPS, these data imply that Sick either mediates or supports Relish activation by Dredd and Imd.

**Table 3:**
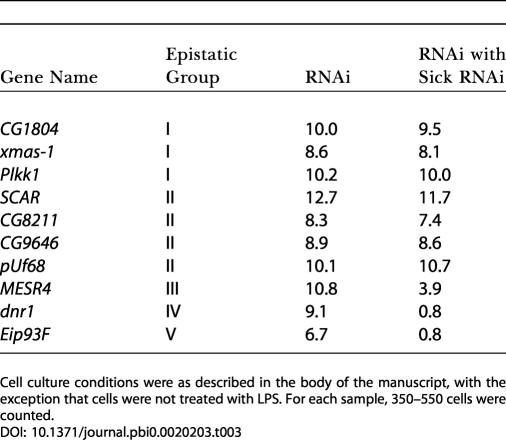
Measurement of the Percent of LacZ-Positive Cells after Treatment With dsRNA against Sick Followed by RNAi for the Individual CDRi

Cell culture conditions were as described in the body of the manuscript, with the exception that cells were not treated with LPS. For each sample, 350–550 cells were counted

### Dnr1 Is a Novel Inhibitor of Dredd

Negative regulators are likely to participate in the circuitry that controls Dredd activation of Relish. The key candidate for action at this level was the single Group IV CDRi gene, CG12489, which showed epistatic relationships consistent with a role in inhibiting Dredd. RNAi of CG12489 induced Dipt-lacZ expression without immune stimulus, indicating that CG12489 normally prevents Dipt expression. As CG12489 inactivation fails to induce Dipt-lacZ in the absence of Dredd or Relish (see Figure 2C), we reasoned that CG12489 normally suppresses Dredd-dependent induction of Dipt-lacZ. We named this gene *dnr1* and discuss its actions more fully below.

**Figure 4:**
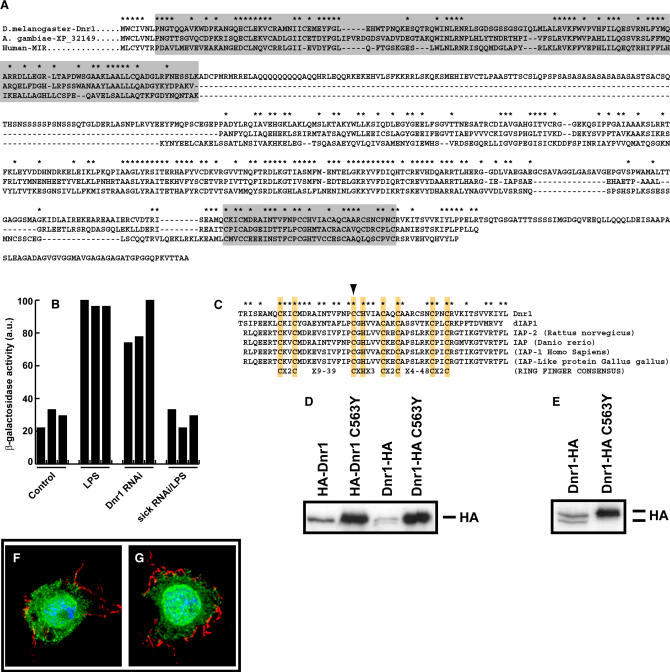
Dnr1 Is a Conserved Inhibitor of Dredd Activity (A) A comparison of the amino acid sequence of Dnr1 with XP_32149 from Anopheles gambiae and human MIR. Shaded regions indicate the N-terminal ezrin/radixin/moesin domain and C-terminal RING finger. Asterisks indicate conserved residues. (B) Measurements of β-galactosidase activity in lysates prepared from Dipt-lacZ in control cells, LPS-treated cells, *dnr1* dsRNA –treated cells, and *sick* dsRNA–treated cells exposed to LPS, respectively. Each experiment was performed in triplicate. (C) Similarity between the RING finger in Dnr1 and other IAPs. Critical residues are shaded. Asterisks indicate conserved residues. (D) Lysates from S2 cells transfected with equal amounts of N- and C-terminally HA-tagged wild-type Dnr1 (lanes 1 and 3, respectively), or N- and C-terminally HA-tagged C563Y Dnr1 (lanes 2 and 4, respectively), and analyzed by an anti-HA Western blot. Residue C563 is critical for RING finger function and is indicated with an arrowhead in (C). (E) Higher resolution of lysates from C-terminally HA-tagged wild-type or C563Y Dnr1. Mutation of the RING finger prevents accumulation of a lower isoform of Dnr1. (F and G) Subcellular localization of HA-Dnr1 transiently expressed in S2 cells treated without (F) or with (G) LPS, with HA in green, DNA in blue, and actin in red.

Dnr1 is a conserved protein with an N-terminal ezrin/radixin/moesin domain and a C-terminal RING finger (Figure 4A). To confirm that Dnr1 inactivation stimulated Dipt-lacZ production, we measured the β-galactosidase activity of lysates from Dipt-lacZ cells treated with *dnr1* dsRNA. Exposure of Dipt-lacZ cells to LPS reproducibly increased Dipt-lacZ production 4- to 5-fold (Figure 4B). Importantly, in three independent experiments, Dnr1 RNAi stimulated Dipt-lacZ production to a similar degree in the absence of LPS. Furthermore, Dipt-lacZ activation in response to LPS was essentially reduced to background levels upon inactivation of *sick.* These findings provide additional support for negative and positive regulation of Relish by Dnr1 and Sick, respectively. While we detected genes with similarity to *dnr1* in many higher eukaryotes, we failed to find a homolog of Dnr1 in *C. elegans.* Interestingly, C. elegans does not rely on an Imd pathway for innate defenses (Kurz and Ewbank 2003). Other RING finger proteins are E3 ubiquitin ligases that target a variety of substrates for proteolytic destruction. The RING finger motif in Dnr1 has greatest sequence homology to the RING fingers found in inhibitor of apoptosis proteins (IAPs; Figure 4C). IAPs are critical inhibitors of caspase activity that ubiquitinate their targets and promote autoubiquitination (Bergmann et al. 2003). Previous reports demonstrated that caspase inhibitors activate their own destruction and that this activity is RING finger mediated (Yang et al. 2000). Consistent with these reports, we observed surprisingly low levels of accumulation of a hemagluttanin (HA)–tagged Dnr1 in transfected cells. A point mutation in a residue critical for RING finger function resulted in increased accumulation of transfected HA-Dnr1 (Figure 4D). We also detected a protein processing event that appears to depend on the RING finger. Upon expression of C-terminally HA-tagged Dnr1, we observed a slightly lower molecular weight isoform, suggesting N-terminal processing of Dnr1 (Figure 4E). The absence of this lower molecular weight isoform in cells transfected with the N-terminally HA-tagged Dnr1 (Figure 4D) is consistent with processing near the N-terminus. This processed isoform was absent in cells transfected with constructs containing the RING finger mutation (Figure 4E). The presence of the RING finger motif, and its apparent role in destabilizing Dnr1, argues that Dnr1 is a caspase inhibitor and that, given its functional role and epistatic position as an inhibitor of Dredd, it is likely to act directly to inhibit this caspase.

### Dnr1 Protein Levels Are Regulated by Dredd Activity

**Figure 5:**
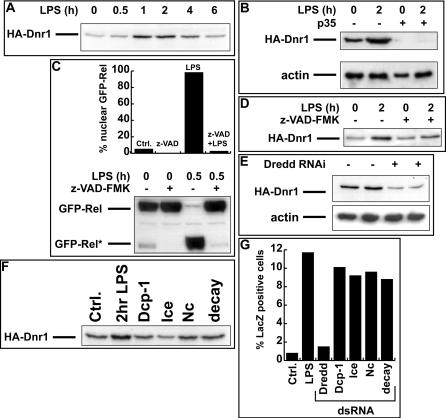
Dnr1 Protein Levels Are Regulated by Dredd Activity (A) Amounts of HA-Dnr1 transiently increase in S2 cells treated with LPS. Anti-HA Western blot of lysates from HA-Dnr1-transfected S2 cells that were incubated with LPS for indicated periods. (B) Anti-HA Western blot of lysates from S2 cells transfected with HA-Dnr1. Coexpression of the caspase inhibitor p35 dramatically inhibits HA-Dnr1 accumulation in the absence (lanes 1 vs. 3) or presence (lanes 2 vs. 4) of LPS. Actin levels are shown as a loading control. (C) Upper panel shows the percentage of cells with nuclear GFP-Relish after the indicated treatments. The lower panel is an anti-GFP Western blot of lysates from S2 cells treated in the identical manner. z-VAD-FMK prevents nuclear accumulation of GFP-Relish and GFP-Relish processing in response to LPS. (D) Anti-HA Western blot of lysates from S2 cells transiently transfected with HA-Dnr1. While 2 h incubation with LPS normally leads to a 4-fold increase (quantified by titration) in HA-Dnr1 (lanes 1 vs. 2), incubation with z-VAD-FMK prevents the accumulation (lanes 3 vs. 4). (E) Anti-HA Western blot of lysates from S2 cells transfected with HA-Dnr1. Cells had been previously incubated with (lanes 3 and 4) or without (lanes 1 and 2) Dredd dsRNA. Results are shown for two independent experiments. Actin levels are shown as a loading control. (F) Anti-HA Western Blot of lysates from S2 cells transfected with HA-Dnr1 shows that prior RNAi against the caspases Dcp-1, Ice, Nc, and Decay does not substantially affect HA-Dnr1 levels (compare with control without LPS). (G) The number of Dipt-lacZ-expressing cells after LPS treatment is greatly reduced after Dredd RNAi, while RNAi against Dcp-1, Ice, Nc, or Decay has no effect.

While LPS had no dramatic effect on the subcellular localization of HA-Dnr1 (Figure 4F and Figure 4G), exposure to LPS had a transient effect on the levels of Dnr1 protein. Addition of LPS caused an increase in HA-Dnr1 levels (Figure 5A), which rose 4- to 5-fold 2 h after treatment with LPS and then gradually declined. Since LPS-dependent processing of Relish by Dredd proceeded in a similar manner (see Figure 3C), we tested whether Dredd inactivation affected Dnr1 protein levels. Cotransfection of the caspase inhibitor p35 along with HA-Dnr1 blocked HA-Dnr1 accumulation (Figure 5B). Similarly, even transient treatment with the caspase inhibitor z-VAD-FMK at concentrations sufficient to prevent Relish processing (Figure 5C) reduced LPS-dependent HA-Dnr1 accumulation (Figure 5D). As these data implicated caspase function in Dnr1 accumulation, we tested the five *Drosophila* caspases represented in our library for their influence on Dnr1 stability. Only Dredd RNAi reproducibly reduced HA-Dnr1 levels (Figure 5E and Figure 5F). Consistent with a role for Dredd as the critical caspase in LPS-dependent Relish activation, of all caspases tested only Dredd inactivation blocked LPS-dependent Dipt-lacZ induction (Figure 5G).

In summary, addition of LPS to S2 cells activates Dredd and stabilizes Dnr1, while inactivation of Dredd by RNAi or caspase inhibitors reduces Dnr1 protein levels. We conclude that Dnr1 protein levels are regulated by Dredd activity. While it is not presently known how Dredd caspase function might influence Dnr1 accumulation, we note that the data are consistent with a negative feedback loop in which Dredd activity promotes accumulation of its own inhibitor, Dnr1.

## Discussion

It was previously recognized that the *Drosophila* macrophage-like S2 cell line responds to bacterial cell wall components with the induction of antimicrobial peptide expression. This model lacks the complexities of communication between tissues that drive the spread of the immune response in larvae (Foley and O'Farrell 2003), but it offers an exceedingly powerful system for identification of mediators of antimicrobial peptide induction. To develop a genetic approach to identify novel signal transduction components, we produced reporter cell lines to follow innate immune signaling and a library of 7,216 dsRNAs representing the conserved genes of *Drosophila* to inactivate genes by RNAi. We focused on a screen for immune response genes in the Imd pathway because it is the less thoroughly understood of the two immune response pathways in *Drosophila.* A central aspect of our strategy for dissection of the pathway was to identify negative regulators as well as positively acting genes. In addition to modulating signal transduction pathways, negative regulators participate directly in signaling when downregulated by the inducing signal. Beyond the inherent importance of this relatively unexplored group of regulators, we were interested in their potential utility as an experimental lever: Identification of inhibitors acting at numerous levels of the pathway provides tools for ordering the action of the positively acting genes in the pathway and vice versa. The experimental approach and strategy proved highly efficient, yielding numerous regulators and defining a cascade of gene action by epistasis. A secondary test for the influence of positively acting genes on the nuclear translocation of Relish and the epistasis order allowed us to narrow our focus to genes that are centrally involved in the immune response. Focusing on the unresolved issue of Dredd regulation, we characterized a negative regulator, Dnr1, that provides a critical check on unwarranted Dredd activity. Our results suggest that Dredd controls Dnr1 stability in a negative feedback loop that restricts Dredd function. Normal activation of the Imd pathway may include release or bypass of this negative feedback loop.

### Categories of Innate Immune Inhibitors

A priori, we considered two roles for inhibitors of the Imd pathway: either suppression of spontaneous activation of immune responses in the absence of infection, or downmodulation of a response to limit or terminate it. We designed screens for both these types of activities. In conjunction with the RNAi screen for dsRNAs that blocked response to LPS, we identified dsRNAs that enhanced the response—EDRis. This phenotype represents a failure to downmodulate the response. In an independent screen without LPS, we identified dsRNAs that resulted in constitutive activation of the pathway—CDRis. Surprisingly, there was remarkably little overlap in the genes identified in these two screens: Of 26 CDRis and 46 EDRis only five were in common. At present, we do not understand the functional underpinnings of the distinctions between inhibitors that sustain quiescence (CDRis) and those that downregulate an ongoing response (EDRis).

Interestingly, groups of inhibitors implicate distinct pathways in immune regulation. For example, of the 17 genes that had the strongest EDRi phenotype, four encode splicing factors and four encode products that appear to interact with RNA. This functional cluster suggests that disruptions to some aspect of RNA processing/metabolism can substantially increase the number of S2 cells that activate expression of the Dipt-lacZ reporter in response to LPS exposure. While we do not know how RNA metabolism contributes to this phenotype, the repeated independent isolation of genes lying in a functional cluster reinforces a conclusion that the process is involved. Several other functional clusters were picked up in our screens. Three genes involved in Ras signaling (MESR4, Ras, and Cnk) were identified as EDRi genes. In addition, we noted weak EDRi phenotypes with three additional Ras signaling components (rolled/MAPK, Dsor1, and Pointed). These findings argue that Ras signaling downregulates responses to LPS. This might represent a negative feedback circuit. However, the finding that MESR4 also has a CDRi phenotype suggests that the Ras/MAPK pathway may also impinge on the maintenance of quiescence.

Several genes involved in cytoskeletal structure or regulation were identified among the inhibitors. Genes encoding tubulin *(α-Tub84D),* a kinesin motor *(Klp10A),* and microtubule-severing function *(CG4448/katanin)* were isolated as EDRi genes. Perhaps an event involving microtubule structures helps limit immune responses. The two cellular actin genes *(Act5C* and *Act42A)* were individually dispensable, but their joint inactivation produced both EDRi and CDRi phenotypes. A regulator of actin function, SCAR, was also identified as a CDRi, and both actin and SCAR CDRi phenotypes fell into epistasis Group II. This suggests that disruption of the actin cytoskeleton in quiescent cells can activate the immune response in a Relish-dependent fashion. Since S2 cells are induced to phagocytose bacteria, and changes in cell shape are induced in response to LPS, it would not be surprising if cytoskeletal functions contribute to immune responses. Indeed, microarray studies showed induction of numerous cytoskeletal components in S2 cells upon incubation with LPS (Boutros et al. 2002). Our findings, however, suggest a different involvement of the cytoskeleton in which it functions to constrain S2 cells, preventing or limiting their innate immune responses.

A previous conventional genetic screen for mutations leading to constitutive action of the Imd pathway in *Drosophila* larvae demonstrated that Relish basal signaling is maintained at a low level by proteosomal destruction of processed Relish (Khush et al. 2002). A Skp1/Cullin/F-box (SCF) component was identified as involved in ubiquitination of the N-terminal Relish domain. We did not include any genes in the category of ubiquitination and proteasome function in our CDRi group. This might mean that this pathway does not influence the cellular responses in the S2 tissue culture system. However, our first round of screening suggested that RNAi to a *Drosophila* F-box resulted in increased basal signaling (unpublished data). This and other tentative indications of involvement of this pathway were either not reproduced or fell below the threshold in retesting. We are left uncertain about SCF contributions to immune induction in our system.

### A GFP-Relish Reporter Line Subdivides DDRi Genes

As in the case of the CDRi and EDRi phenotypes, our screen for DDRi phenotypes identified numerous genes falling into functional categories. One potential limitation of our approach for identification of DDRi is that some genes required for ecdysone maturation may be selected as immune deficient. Additionally, one of the largest functional categories was genes involved in translation and included four ribosomal proteins, three initiation factors, two amino acyl-t-RNA synthases, and an elongation factor. It seems likely that RNAi of genes in this category affects translation of the Dipt-LacZ reporter, as opposed to affecting modulation of signaling events. To cull our collection of DDRis of such indirect modulators of the response, we developed a secondary screen that does not rely on de novo gene expression. Based on the previously described phenotypes of Imd pathway members, we reasoned that inactivation of the core components transducing the signal would compromise activation of the Relish transcription factor. To identify DDRi dsRNAs that prevented Relish activation, we prepared a GFP-Relish reporter cell line and rescreened DDRi dsRNAs for loss of GFP nuclear translocation in response to LPS.

In addition to confirming a requirement for Dredd and PGRP-LC in Relish activation, we implicated a proteosomal regulatory subunit Dox-A2 and identified a novel gene *sick* as involved in Relish nuclear translocation in response to LPS. Although cells treated with *sick* dsRNA failed to mount an immune response, the cells were otherwise healthy through the course of the experiment. Dox-A2 RNAi reduced the survival of cells and was effectively lethal within a few days of the scoring of the immune response. We conclude from this that Sick and Dox-A2 contribute to the central signal transduction process, but it is presently unclear whether Dox-A2 has a significant specific input or if its effects are secondary to a global effect on cell viability.

It is notable that only two DDRi genes passed our secondary screen based on GFP-Relish localization. Does this mean that all the other DDRis are not really involved? While we have not yet analyzed all these genes, we suspect that many of them will modify the Imd pathway, either impinging on the pathway at a point beyond Relish translocation, or quantitatively or kinetically modifying Relish translocation in a manner that we did not detect in our screens. Insight into this issue is likely to be derived from further epistasis tests that might place some of these DDRis in the signaling pathway.

### An Epistatic Network to Position CDRi and DDRi Genes

We identified an unprecedented large number of immune response inhibitors (CDRi genes) in our screens. As there are diverse steps within and potentially outside the Imd signaling pathway at which the CDRi inhibitors might act, we sought to position their actions with respect to known Imd pathway functions by RNAi epistasis tests. By sequential inactivation of known Imd pathway components and CDRi gene products, we tested whether constitutive activation of immune reporters by CDRi dsRNAs depends on steps in the signal transduction pathway. In this way, we defined five distinct epistatic categories of CDRi gene products. The four CDRi genes that continue to activate immune responses despite inactivation of Imd, Dredd, or Relish are likely to act on signal-transduction-independent factors that maintain transcriptional quiescence of Dipt. The largest group of CDRis (12) depends on Relish function but not on upstream activators of Relish. These are likely to include two types of regulators: one type that sets the threshold of response so that basal activity of Relish does not trigger pathway activity, and a second type that contributes to suppression of Relish activity. The latter type of regulator might include inhibitors that impinge on the late steps in the signal transduction cascade. For example, genes whose normal function inhibits the activity of the full-length Relish transcription factor might be required to make the pathway activator dependent, and these would be found in this category. The remaining upstream epistasis groups that rely on additional signal transduction components are strongly implicated as significant contributors to the immune induction pathway. As all of the CDRis induced robust immune responses in the absence of ecdysone (unpublished data), we propose that the CDRis have their input into the Imd pathway at a level that is the same or lower than the level of the input from ecdysone. Given that this is true for all five epistatic groups of CDRis, the result suggests that ecdysone has its input at an early level of the Imd pathway.

The identification of five epistasis groups of inhibitors also provides reference points for a second round of epistasis tests that position novel DDRi genes within the Imd pathway. We used this approach to show that the novel DDRi *sick* is required for constitutive activation of the responses by inactivation of CDRi genes in Groups III, IV, and V genes but not for the action of CDRi Group II or Group I genes. If we assume a simple linear pathway, this would indicate that Sick functions upstream of Relish and downstream of Imd and Dredd. It is noteworthy that the epistatic data are consistent with molecular data indicating that Sick is required for Dipt-lacZ induction and the nuclear translocation of Relish in response to LPS. This combination of phenotypic, epistatic, and molecular data argues for participation of Sick in the regulated activation of the Relish transcription factor.

### Dnr1 Prevents Ectopic Dredd-Dependent Relish Activation

One epistatic group struck us as particularly interesting. While Dnr1 inactivation caused ectopic Dipt-lacZ expression, simultaneous loss of Dredd or Relish restored cells to their resting state. These data indicate that the wild-type function of Dnr1 is to prevent Dredd-dependent activation of Relish. Consistent with this hypothesis, we identified a C-terminal RING finger in Dnr1 with greatest similarity to the RING finger motifs observed in the C-terminus of IAP proteins. In addition to regulating caspase activity, IAPs also regulate their own stability through ubiquitin-mediated proteolysis. Similarly, we observed that mutation of a critical RING finger residue greatly stabilized Dnr1. These features suggest that Dnr1 is a caspase inhibitor, suggesting that it might act directly to inhibit Dredd activity.

**Figure 6:**
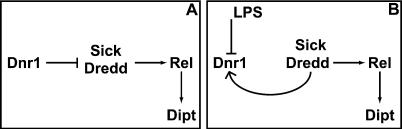
A Schematic of the Proposed Relationships of the Novel Immune Regulators, Sick and Dnr1, to Dredd and Rel Pointed and blunt arrows indicate activation and inhibition, respectively. Both Sick and Dredd are required for translocation of Rel to the nucleus and for activation of Dipt expression and they are consequently positioned upstream of Rel as activators. In the absence of Sick or Dredd, Dnr1 function is not needed to maintain pathway quiescence. Thus, Dnr1 is ordinarily required to either inhibit Sick and Dredd functions or to negate their actions, and we have indicated these regulators as being downstream of Dnr1 (A). Although we have no epistatic data that separates the action of Sick and Dredd, Dredd appears to directly cleave Rel and is hence likely to be immediately upstream of Rel. Sick might function in conjunction with Dredd or as an activator of Dredd. Since *dnr1* RNAi does not enhance the response to LPS, we suggest that its inhibitor activity is either repressed or bypassed upon exposure to LPS. Consequently, we have shown that treatment with LPS counteracts Dnr1-dependent Dredd inhibition (B). We do not mean to preclude other actions of LPS that might contribute to induction, but it is notable that inactivation of Dnr1 is sufficient to activate signaling. Finally, we have shown that Dnr1 levels are affected by Dredd, and we have indicated this with a positive feedback arrow.

We observed that exposure of cells to LPS transiently stabilized Dnr1 and that this stabilization directly paralleled the period of Dredd-dependent Relish processing. This suggested to us that Dnr1 stability and accumulation might be regulated by its target, Dredd, a regulatory connection that could establish a negative feedback loop. We confirmed that Dredd activity is required for accumulation of Dnr1. These results suggest that Dredd modulates a RING-finger-dependent Dnr1 destruction pathway (Figure 6).

Our results are consistent with a feedback inhibitory loop where Dredd activity promotes accumulation of its own inhibitor (Figure 6); however, it is not clear under what circumstance this loop functions. Since Dnr1 inactivation did not enhance Dipt-lacZ production by LPS, we propose that Dnr1 inhibition of Dredd is suppressed or bypassed by LPS treatment and that Dnr1 is not essential for downregulation of an ongoing response. Further, as suppression of Dnr1 by RNAi is sufficient to activate immune responses, Dnr1 functions in the absence of induction and this function is required for quiescence. Thus, LPS inactivation of Dnr1 function ought to be sufficient to trigger Dredd-dependent cleavage of Relish in the Imd pathway, and it could make a significant contribution to pathway activation.

In summary, a new and powerful screening approach has provided many candidate regulators of the Imd pathway of the innate immune response, and we suggest that the newly identified contributors Dnr1 and Sick will govern central steps in the regulatory cascade that activates the Relish transcription factor. While our analysis has led to a focus on these two regulators, we suspect that other genes among those isolated will also make important direct contributions to the Imd pathway. Furthermore, some of the groups of genes falling into functional clusters are likely to define physiologically relevant inputs into the induction pathway.

## Materials and Methods

### 

#### Generation of dsRNA library

A library of DNA templates bearing the T7 RNA polymerase promoter at each 5′ end was prepared from genomic DNA in a two-step PCR protocol. In the first round of PCR, targeted regions of DNA were amplified using gene-specific primers (18–22 nucleotides) with a 5′ GC-rich anchor (GGGCGGGT). Primers were designed to amplify a region of nonintronic genomic DNA between 250 and 800 bp with minimal sequence overlap to all other amplimers. Templates from the first step were amplified in a second round using a universal primer containing the T7 RNA polymerase promoter sequence followed by the GC-rich anchor TAATACGACTCACTATAGGGAGACCACGGGCGGGT. dsRNA was generated from templates in in-vitro transcription reactions for 6 h at 37 °C. In vitro transcription products were annealed by heating to 65 °C and slowly cooling to room temperature. All products were tested for yield and size by gel electrophoresis, with 97% giving satisfactory results.

#### Generation of stable cell lines

Dipt-lacZ cell and copper-inducible GFP-Relish stable S2 cell lines were generated according to the Invitrogen *Drosophila* Expression System Protocol using hygromycin B (Invitrogen, Carlsbad, California, United States) as a selection marker. The Dipt-lacZ plasmid has been described previously (Dimarcq et al. 1997). To prepare N-terminally GFP-tagged full-length Relish, the stop codon in enhanced GFP (EGFP) was replaced with a Not site, and EGFP was cloned into pUAST as an EcoRI/NotI fragment. Full-length Relish cDNA was fused in frame to the EGFP coding sequence as a NotI/XbaI fragment, and the entire sequence was confirmed by sequencing. GFP-Relish was cloned into pMT/V5-HisB as an EcoRI/XbaI fragment to allow copper-inducible expression of GFP-Relish under control of the metallothionine promoter.

#### Cell culture

S2 cells were plated into glass-bottomed 96-well microplates (BD Biosciences Pharmingen, San Diego, California, United States) with 40,000–50,000 cells in 200 μl of Schneider's *Drosophila* medium (GIBCO, San Diego, California, United States) supplemented with 10% heat-inactivated fetal calf serum, penicillin, streptomycin, and hygromycin per well. dsRNA was added to each well at a final concentration of 10 μg/ml. Cells were cultured for 4 d at 25 °C and incubated an additional 24 h in 1 μM 20-hydroxyecdysone (Sigma, St. Louis, Missouri, United States). LPS (Calbiochem, San Diego, California, United States) was added at a final concentration of 50 μg/ml for 12 h. RNAi protocols were as previously described (Clemens et al. 2000).

#### β-galactosidase assays

To measure β-galactosidase in S2 cells, medium was aspirated from the wells, and cells were fixed in 0.5% glutaraldehyde in PBS for 30 s. Cells were then incubated in X-Gal staining buffer overnight at 37 °C (10 mM phosphate buffer [pH 7.2], 150 mM NaCl, 1 mM MgCl_2_, 3.5 mM K_3_Fe(CN)_6_, 3.5 mM K_4_Fe(CN)_6_, and 0.2% X-Gal in DMF). β-galactosidase activity assays were performed as described previously (Dimarcq et al. 1997).

#### Microscopy, immunofluorescence, and image processing

β-galactosidase induction in S2 cells was observed with a Leica (Wetzlar, Germany) IMRB microscope. Immunofluorescent images were taken on an Olympus (Tokyo, Japan) IX70 driven with DeltaVision software (Applied Precision, Issaquah, Washington, United States). S2 cells were deposited on Superfrost Plus Gold slides (Fisher Scientific, Hampton, New Hampshire, United States) for immunofluorescence. Cells were fixed for 10 min in 4% formaldehyde (Sigma). Tubulin was detected with mouse anti-α-tubulin (Sigma). DNA was visualized with Hoechst 33258, and actin was visualized with rhodamine-coupled phalloidin (both from Molecular Probes, Eugene, Oregon, United States). Images were processed with Adobe Photoshop 5.5, and figures were assembled with Adobe Illustrator 9.0.

#### Western blotting

Dnr1-expressing vectors were prepared by cloning Dnr1 cDNA into pAc5/V5HisA (Invitrogen). The C365Y mutant form of Dnr1 was prepared with the Stratagene point mutation protocol using a TTCAATCCGTACTGTCACGTC sense primer and a GACGTGACAGTACGGATTGAA antisense primer. The mutation was confirmed by sequencing. For experiments with z-VAD-FMK, S2 cells were incubated in 100 μM z-VAD-FMK for 4 h at room temperature. Cells were harvested by centrifugation at 1,000 *g* for 3 min, washed in PBS and lysed on ice for 10 min in lysis buffer (0.5 M HEPES [pH 7.5], 150 mM NaCl, 5 mM EDTA, 0.2% NP40, PMSF, leupeptin, pepstatin, NaF, and microcystine LR). Lysate was spun for 10 min at maximum speed, and the supernatant was added to sample loading buffer. Samples were separated by SDS-PAGE and analyzed by Western blotting. Anti-GFP antibody was purchased from BabCO (Richmond, California, United States), and HA and actin antibodies were purchased from Sigma.

**Table 1:**
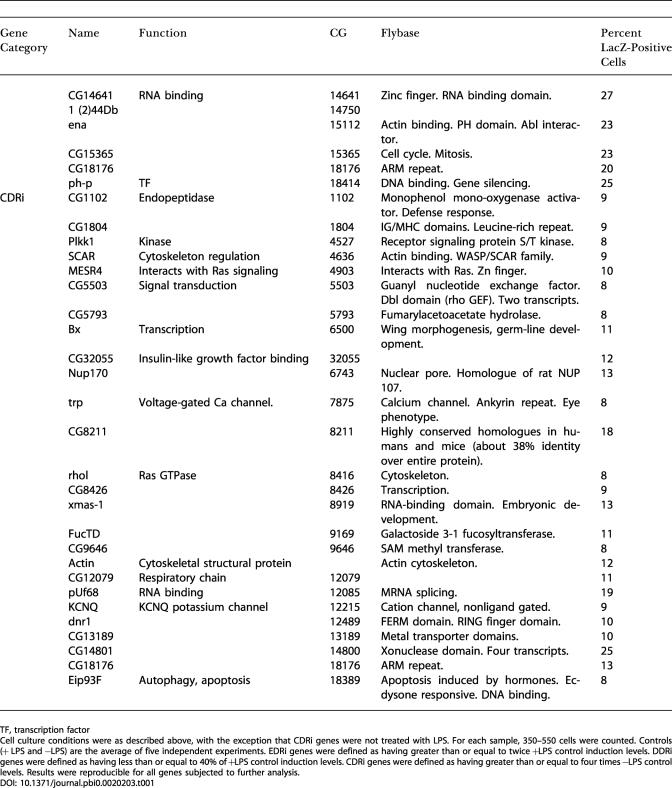
Continued

**Table 1:**
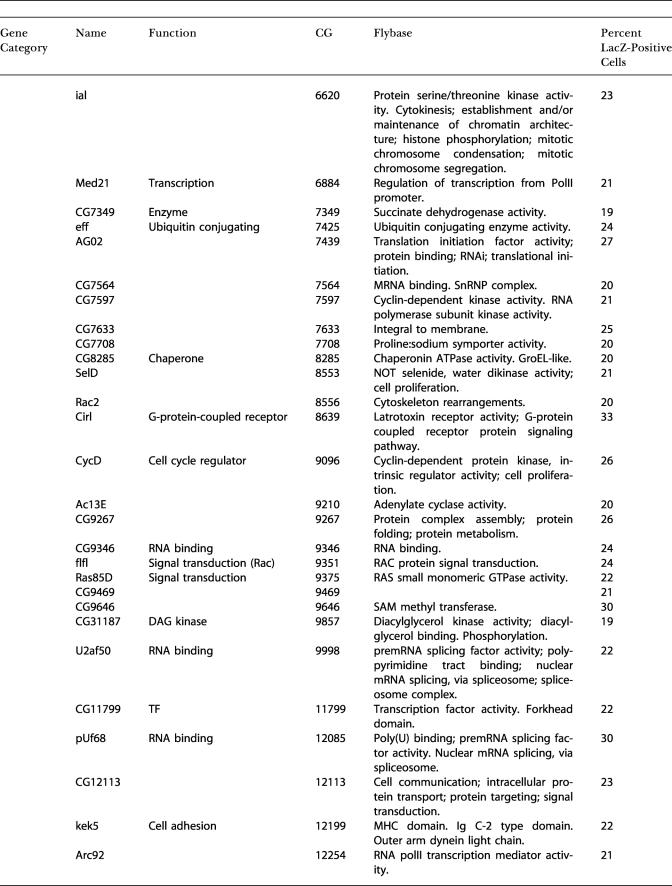
Continued

**Table 1:**
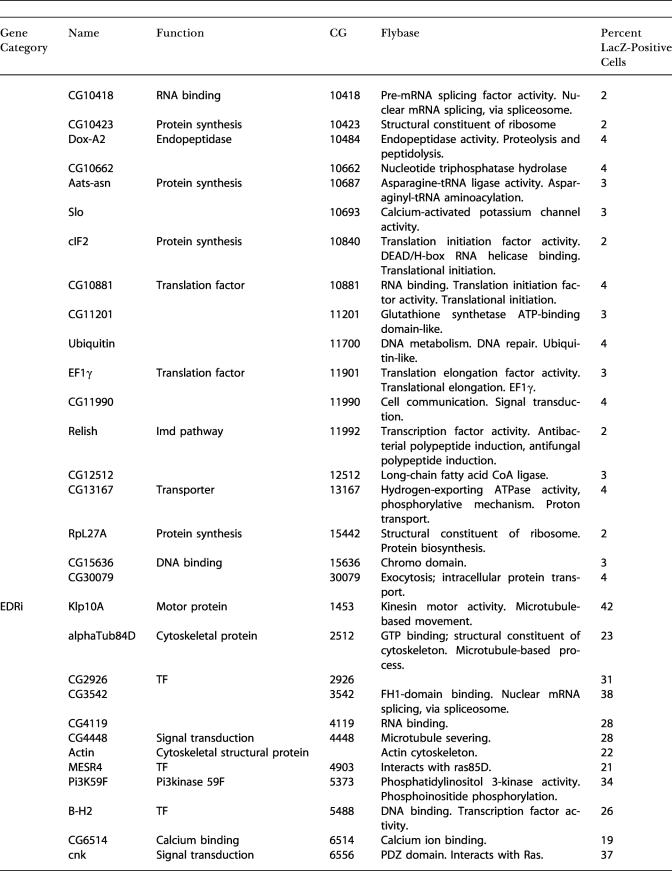
Continued

## Abbreviations

CDRi: constitutive defense by RNAi
DDRi: decreased defense by RNAi
Dipt: Diptericin
Dnr1: Defense repressor 1
dsRNA: double-stranded RNA
EDRi: enhanced defense by RNAi
EGFP: enhanced green fluorescent protein
GFP: green fluorescent protein
HA: hemagluttanin
IAP: inhibitor of apoptosis protein
Imd: Immune deficiency
LPS: lipopolysaccharides
MAPK: mitogen-activated protein kinase
PGRP-LC: peptidoglycan recognition protein LC
RNAi: RNA interference
SCF: Skp1/Cullin/F-box
*sick*: *sickie*
